# Compliance in recreational fisheries: Case study of two blue swimmer crab fisheries

**DOI:** 10.1371/journal.pone.0279600

**Published:** 2023-01-06

**Authors:** Jade Lindley, Liam Quinn

**Affiliations:** 1 Law School and The Oceans Institute, The University of Western Australia, Perth, Australia; 2 Law School, The University of Western Australia, Perth, Australia; Shiv Nadar University, INDIA

## Abstract

Comparing two Australian regions, Western Australia (WA) and South Australia (SA), this research investigates official noncompliance datasets of recreational blue swimmer crab *(Portunus armatus)* fishing between 2009 and 2019. These recreational fisheries in both jurisdictions are license-free and therefore participating fisher information is limited. Analyses provide a glimpse at the (noncompliant) fisher population profiles against the application of management strategies. The data provide (1) an evidence-base to optimize regulatory strategies by balancing education and enforcement activities with recreational fisher enjoyment. The results of this research enable application within and beyond these fisheries and jurisdictions; and (2) drawing from the criminology discipline, deterrence theory offers insight to enhance compliance tools. Further, it shows the importance of a multi-disciplinary approach to assessing compliance and identifies some practical approaches to data collection that can be readily undertaken to assist with more detailed analysis and enhance compliance strategies.

## Introduction

The Western Australian (WA) Department of Primary Industries and Regional Development (DPIRD) and the South Australian (SA) Primary Industries and Regions South Australia (PIRSA) each have primary responsibility to conserve, sustainably develop and equitably manage fish resources. Both jurisdictions manage blue swimmer crab (*Portunus armatus*) fisheries accessed by commercial, recreational and customary fishers. In 2016 the international third-party certification body, the Marine Stewardship Council (MSC) certified its first joint commercial and recreational fishery, the iconic WA Peel-Harvey Estuary blue swimmer crab fishery [1, 2]. Maintaining this as a sustainable recreational fishery is a high priority for DPIRD and the WA recreational fishing sector.

Non-compliance herein is defined as actions that contravene the legal statutes that govern each fishery and relevant penalties are suitably applied for minor and serious matters, as appropriate. Neither jurisdiction require a recreational license for the blue swimmer crab fisheries, which means there is no framework upon which to base recreational surveys targeting crabs making fisher demographics and fishing activities difficult to obtain. Each jurisdiction collects data on fishers during interactions with patrolling officers where there are instances of noncompliance. The large spatial areas of the fisheries make it impractical to closely monitor all recreational fishing activity. Increasing patrolling effort or introducing additional restrictions would no doubt enable increased protection of stocks; however, this would decrease recreational fisher enjoyment. Achieving a balance between enjoyment and regulation, relies on compliance among participants engaged in these fisheries.

According to the Australian Fisheries National Compliance Strategy, ‘optimal compliance’ strikes an acceptable balance between a reasonable cost without compromising the integrity of management and resource sustainability [3]. In this setting, this research aims to contribute to the existing understanding of optimal compliance and determine how to aptly apply enforcement and education strategies, without reducing fisher enjoyment and participation in the fishery. By analyzing blue swimmer crab noncompliance datasets held by DPIRD and PIRSA, and drawing on criminological theory, trends can inform regulatory frameworks for enhanced governance and innovative approaches to optimize fisher education and enforcement strategies. The results from this study may be applicable beyond this project’s fisheries and jurisdictions.

Social science research is increasingly being used to inform fisheries management strategies. Recent studies consider the human dimension of recreational fishers relevant to the blue swimmer crab fishery in Australia, including perceptions of environmental management strategies to enhance recreational compliance [4]; local fisher knowledge and the perceptions of historical abundance trends [5]; the network of communication regarding the blue swimmer crab fishery [6]; and managing nocturnal fisheries compliance of the blue swimmer crab fishery [7]. Following a brief background on the blue swimmer crab fisheries involved in the present study, the remaining background sections survey recent social science research into perceptions of compliance, as well as managing compliance. This project builds on existing multidisciplinary approaches to understand ways to address noncompliance in this fishery.

### Blue swimmer crab recreational fisheries in WA and SA

Blue swimmer crabs *(Portunus armatus)* exist across Australia. The recreational fishing sector is popular in both SA and WA, with recreational fishers harvesting a large proportion of the total catch of blue swimmer crabs [8, 9]. Blue swimmer crabs are distributed along the entire Western Australian coast, mainly between Nickol Bay and Dunsborough, with the vast majority of recreational fishing taking place in the estuaries and coastal embayments from Geographe Bay to the Swan River and Cockburn Sound [8]. The Peel-Harvey Estuary is an area of approximately 136 km^2^ that provides significant habitat for blue swimmer crabs. Blue swimmer crabs are found throughout the inshore waters of SA, with fishing zones in Spencer Gulf, Gulf St Vincent and West Coast [10]. This research focuses solely on the recreational fisheries of two regions: the recreational Peel-Harvey blue swimmer crab fishery in WA and the recreational blue swimmer crab Gulf St Vincent and Spencer Gulf fishery in SA, herein referred to as the WA fishery and the SA fishery, respectively.

In 2016, the Peel-Harvey blue swimmer crab fishery became the first Marine Stewardship Council (MSC) certified recreational fishery [8]. MSC certification is based on the three broad principles of: sustainability of the fishery, the environmental impact of fishing, and governance and management of the fishery. The latter principle involves an assessment of the fisheries’ compliance and enforcement regime [2].

The breeding stock of each fishery is an important element of the management strategy in place. Increased stock availability can increase fishery catch allocations available to both commercial and recreational fishers, while ensuring the fishery stock is maintained sustainably. The breeding stock status in the Peel-Harvey Estuary in WA is assessed as being adequate and sustainable based on commercial catch data [11]. Recreational catch estimates are infrequent and have typically been in the range of 35-49t with 95% confidence interval [11]. The availability of crabs is highly dependent on environmental conditions in the estuary which affects the growth of crabs. Meanwhile in SA, recreational stock assessments are no longer maintained, though the commercial sector accesses approximately 70 percent of the estimated quota, the remainder sustainably allocated among recreational and Indigenous fishers [9]. Understanding the stock situation is critical to fishery management and contributes to how regulatory strategies are applied. Difficulties in obtaining accurate data on catch and effort for such large recreational fisheries makes management harder, and more uncertain. This uncertainty can place additional pressure on fishers and regulators to ensure governance arrangements in these fisheries are both practical and generally complied with.

Overarching legislation provides governance for the fishery in each jurisdiction. In WA, the *Fish Resources Management Act 1994* and the Fish Resources Management Regulations 1995 provide a comprehensive suite of fishery management tools to manage blue swimmer crab fishing in the Peel-Harvey estuary. In SA, the *Fisheries Management Act 2007* and the Fisheries Management (Blue Crab Fishery) Regulations 2013 are the overarching legislations for the blue crab fishery. Table 1 provides further details.

**Table 1:** Summary of blue swimmer crab management tools, Western Australia and South Australia, as at 2022.

Management Tool	Purpose	SA Implementation	WA Implementation
Catch Limit	To limit catch taken per fisher, sharing the resource and contributing to the sustainable management of the fish stock.	20 –combined limit with Sand Crabs. Daily boat limit when 3 or more people are crabbing on board: 60 –combined limit with Sand Crabs.	Ranges from 5–20* based on fishing location. In the West Coast bioregion the limit is 10. A boat limit of 20 crabs per licensed fisher also applies**
Size Limit	Allow fish to reach maturity to complete their breeding cycle.	11 cm measured across the carapace from the base of the largest spines.	Crabs taken must be at least 127mm across the widest part of the carapace.
Spatial Closures	To create sanctuary areas of unfished habitat, for stock, habitat or ecosystem protection.	Nil.	Cockburn Sound is closed to crabbing. No crabbing in any Marine Park Sanctuary Zone, but no SZs in the Peel-Harvey Estuary.
Temporal Closures	To remove fishing effort during certain times of the day or year.	Nil.	Closed from 1 September to 30 November (from 2019, prior to that closed 1 September to 31 October).
Additional Breeding Stock Protections	To enhance the breeding stock by requiring vulnerable and valuable fish to be returned to the water unharmed.	Females with external eggs are totally protected and must be returned to the water immediately.	Egg-carrying (’berried’) females must be returned to the water immediately, before attempting to catch another crab
Gear Design Restrictions***	To minimise harmful effects to fish and ecosystems. To allow targeting of certain size classes. To decrease catch per unit effort by making fish harder to catch.	Baiting: When using hoop nets or drop nets in marine waters (including from a jetty or boat) you cannot use any type of meat, chicken or other poultry in hoop nets or drop nets. See the bait and berley guidelines for more information.	Dredges or rakes, obstructions, nets, poisons, explosives, traps, pots, set-lines, hooks and sharp implements and commercial fishing gear are prohibited in the fishery.
Reporting Requirements	To enable better understanding of catch and effort by those managing the fishery and the fish stock it depends on.	Nil.	Nil.
Restriction on sale & barter	To maintain resource equity with commercial fishers. To deter people from taking catch for reasons other than enjoyment of the fishing experience and consumption of fresh seafood.	It is an offence for recreational fishers to sell or trade their catch.	It is illegal for recreational fishers to sell or barter their catch.
Licence requirements	To create reciprocal obligations between those with a fishing right, and those who pay for fishery management. To identify people authorised to undertake fishing activities. To provide revenue to help cover the costs of management.		If a powered boat is used to fish for crabs or to reach the fishing location, at least one person on board needs a Recreational Boat Fishing Licence.
Possession restrictions	Restrictions applied to how catch is stored and transported when fishing is not underway.		All uncooked crabs must be kept in whole form, unless being prepared for immediate consumption.

*there are also limits on the number that can be female in some locations

**each individual is only allowed the daily bag limit and must hold their own boat licencse

***there are also limitations on the amount of gear that is permitted for use at any one time

Both WA and SA engage in educational campaigns to encourage compliance among participating fishers. Examples in WA include large multi-lingual temporary banners erected near popular fishing spots, permanent signage at beach access points and car-parks, mobile illuminated trailer signs, dedicated educational outreach programs, social and digital medial detailing the rules, for instance a *Rules App* published by Recfishwest, and the DPIRD Fisheries website. While examples in SA include permanent signage at popular beach access points and carparks; illuminated messaging trailer signs strategically positioned in popular fishing locations; via social and digital medial including a recreational fishing app, and the PIRSA website with up-to-date fisher information; and Fisheries Officers and Fishcare Volunteers distribute crab measuring gauges and blue crab information brochures.

Additionally, in WA, strategic communication using media articles promote sustainable fishing messages and create *general* deterrence following newsworthy apprehensions and court outcomes. Furthermore, SA expands the messaging of court outcomes to include social media platforms. Collectively, these instruments govern the fishery and promote compliance.

### Perceptions around compliance

Fisher noncompliance is a pervasive issue that contributes towards potentially irreversible harm to fishing stock the world over [12, 13]. Extensive research has been conducted across a range of global fisheries that considers fishers’ perceptions and attitudes towards compliance from an economic, social and ecological perspective. While it is well known that fishers will comply even when there are opportunities and incentives not to do so [14]; it has also been found that non-compliance amongst fishers is common too. For example, a study in the United Kingdom found that despite fisher awareness of local no-take areas, non-compliance was common in no-trawl areas [15]. Slater et al.’s [16] Tanzanian study on perceptions of non-compliant fishing and marine ecosystem health found a strong culture of noncompliance. Another study looking at incentives and sanctions in Spain, concluded that greater value was placed on penalizing as a default, rather than rewarding compliance [17]. A south-eastern African study found that increasing costs and benefits transparency with fishers would increase compliance for selected fisheries regulations [18]. Another study considered psycho-social characteristics of recreational fishers to assess the relationship between two factors: ecological values and personality types and recreational fisheries noncompliance [19]. A US study focused on economic and normative factors that motivate compliance decisions, found that regulatory violations increase when detection likelihood, and reliance on gear are both low [20]. A Canadian study found that despite regulation declaring conservation areas, noncompliance continued in these zones [21]. Finally, a study looking at blue swimmer crab fishers in WA only, concluded that both recreational and commercial fishers were concerned with compliance with recreational fishers favoring increased length of the seasonal closure, and commercial fishers suggested recreational shore-based fishing licenses should be introduced [22]. While the specific mechanisms in place to set catch limits, manage quotas and monitor and enforce regulations differ between regions, collectively, the results from these studies show that while fishers are aware of regulations a culture of non-compliance is common, but of variable prevalence and impact.

### Noncompliance in WA and SA

Recreational fishing is a cherished pastime worldwide and forms an important part of culture in many societies; however, activity deemed ecologically unsustainable must be appropriately dealt with. Increasingly, fisheries management agencies are managing beyond sustainability, as their oversight extends to managing competing allocations and rights between different groups and external pressures such as environmental stressors and market forces. As such, a range of management strategies may be adopted to control these fisheries, including licenses for fishers and fishing vessels; effort and gear restrictions; spatial and temporal closures; restrictions on sale and supply of fish and fish products; quotas and catch limits; management regimes for specific fisheries; protected species and reserve areas; and a range of penalties for breaches. Restrictions are often underpinned by stock assessments, which rely in part on reported commercial catches and surveyed recreational users. In license-free fisheries, assumptions and estimates may need to be heavily relied upon. While estimates are usually conservative enabling some margin of error, high levels of non-compliance can invalidate assumptions about catch and effort and undermine sustainable management goals. Therefore, to maintain sustainable, equitable fisheries, effort to limit non-compliance is essential.

The large number of illegal fishing incidents of blue swimmer crabs in the Peel-Harvey Estuary (WA) has been a major concern for over a decade [see 2, 23–31]. High rates of prosecutions and infringements are attributable, in part, to a strong compliance presence and the need to create *general* deterrence; it is also indicative of the extent of the noncompliance problem. The Peel-Harvey Estuary blue swimmer fishery has been identified as having the highest level of non-compliance in a WA fishery; taking undersize crabs in the Estuary has comprised around 20% of all recreational fishing offences in WA annually [32].

The prominence of taking undersize crabs in the context of non-compliance in WA blue swimmer crab fisheries is also reflected in media reports. While not all noncompliance is reported in the media, on occasion, cases are reported to publicly deter would-be noncompliers. For example, in December 2018, a Mandurah woman found 15 cooked undersized crab shells, reportedly a common occurrence for her [33]. In March 2018, three men in Mandurah were found guilty of possessing 4, 5, and 39 undersized crabs, respectively [34]. Other non-compliant behaviors have also been reported in the media. In November 2015, it was reported that commercial blue swimmer crab fishers in the Peel-Harvey and Mandurah regions had complained of thefts from crab pots and damaged fishing gear, which was attributed to recreational fishers struggling to catch crabs early in the season [35]. More recently, 10 people were reportedly prosecuted for taking large numbers of undersized blue swimmer crabs in the Mandurah region. A group of four of these fishers were reported to have taken over 100 crabs which is well over the daily bag limit, with 96 of the crabs undersized [36].

High levels of non-compliance for recreational fishing of blue swimmer crabs in SA have also been reported in the media, with noncompliance activities identified including undersize crabbing and exceeding bag and boat limits [37]. In September 2018, a group of three people had been found with 100 undersized blue swimmer crabs [38]. More recently, north of Adelaide a man allegedly possessed 126 blue swimmer crabs, 124 of which were undersized [39].

### Deterring noncompliance in WA and SA fisheries

Leveraging insights from deterrence theory in criminology provides an evidence-based avenue to crime prevention in fisheries. Deterrence theory is based on the premise that people will commit crimes (or fail to comply) if the opportunity exists, unless punishments are *swift*, *certain* and appropriately *severe* [40]. As such, approaches to deter noncompliant behaviors must outweigh the benefits. The fear of punishment can be a suitable deterrent, however it rests on suitable capacity and capability of law enforcement interrupting the activity and the courts imposing a sufficient penalty, which is not always viable, especially in under-resourced fisheries management [40, 41]. Further, *specific* (individuals) or *general* (community at large) deterrence can be applied to offenders through penalties and overarching responses to crime. A deterrence theoretical underpinning forms the basis to responses to noncompliance activities suggested in this research. Applied specifically to the WA and SA blue swimmer crab fisheries, deterrence theory can assist in strategizing the most appropriate responses to best optimize compliance [3].

## Materials and methods

The WA and SA regulators, DPIRD and PIRSA respectively, collect compliance data as part of their operational business. Incidents of noncompliance are recorded by patrolling officers in realtime into data management system(s) called Electronic Patrol Reports and eBrief in WA and Fisheries and Aquaculture Collection Tool (FACT) and eBrief in SA. The data recorded informs operational activity and deidentified extracts of the datasets held can be used to respond to internal, public and ministerial requests. However, targeted analyses of these datasets have been until now, absent in the public domain. This section provides an overview of the datasets that form part of a larger ongoing project, funded by the Australian Government to analyze emergent trends and understand how best to balance compliance and participant enjoyment in a license-free, recreational fishery to ensure the fishery remains sustainable and viable into the future. For the purposes of that project, cleansed and deidentified noncompliance datasets were provided by regulators to researchers at the University of Western Australia for analysis. Ideally, results can inform other fisheries in these and other jurisdictions, where relevant and comparable.

To assist regulators with improving compliance in these fisheries, the project involved analysis of anonymized datasets held by DPIRD and PIRSA. Each jurisdiction’s data holding dates prior to the period analyzed (2009 through 2019), however for reasons of completeness and reliability, data collected prior to 2009 were excluded. Deidentified extracted data were cleansed prior to handover for analysis. The process of analysis underwent the following activities: data collation, generating logic formulas to produce time series results output aggregated at the monthly and annual level, conducting basic descriptive analyses, and producing time series graphical output to visualize emergent trends.

Data collation involved organizing the datasets such that they were comparable across jurisdictions and suitable for conducting descriptive analyses with. For example, the age of offenders in the WA dataset was converted from age in days to age in years. Similarly, offence date and time variables were separated and converted into the appropriate data type. Data collation also involved producing additional columns that captured the output of logical arguments spanning multiple existing data columns. Logic formulas were then written and executed to produce time series output for descriptive analysis. For example, a logic formula commonly used was ‘COUNTIFS’, to calculate the number of unique offences when multiple arguments were satisfied, including aggregating offences at the monthly and annual level.

The time series results output was captured in separate data tables for each analysis, which enabled simple descriptive analyses such as totals, proportions, averages, and standard deviations to be calculated. Time series figures were then produced from the results output for visual inspection of emergent trends. Microsoft Excel was used in each of these tasks due to the simplicity of the descriptive analyses conducted and the visual interface of this software aiding instantaneous and ongoing verification of the results output.

## Results and discussion

While both regulatory agencies, DPIRD for WA and PIRSA for SA have been collecting noncompliance data for these fisheries since at least 2009, analyzing data collected has not been done independently. As such, there exists a suite of data that provides insight into non-compliers participating within these fisheries. This information can contribute to minimize cost to the regulators by refining enforcement activities and maximize effectiveness of education campaigns. While operational staff understand these fisheries, the analysis confirms and establishes an evidence base from which resource and sustainability decision-making can be made with confidence.

### Results

**Fig 1:**
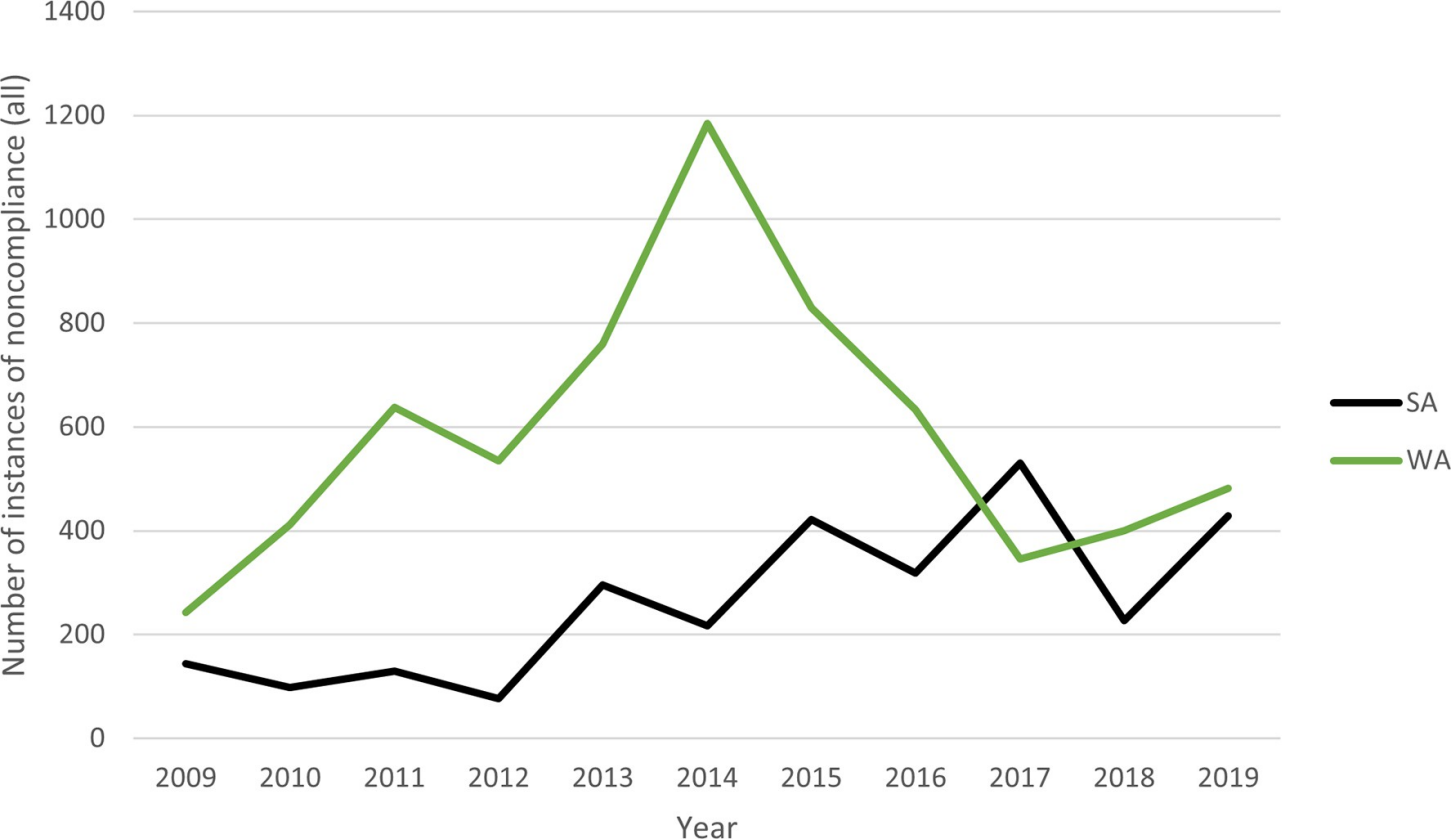
Number of instances of noncompliance (all) in Western Australia and South Australia (2009–19).

During the 11 years between 2009 and 2019, WA recorded 6,462 incidents of non-compliance relating to the Peel-Harvey blue swimmer crab fishery, while SA recorded 2,884 incidents of non-compliance across the same period for the blue swimmer crab fishery. Fig 1 shows a breakdown by year for WA and SA. While overall, WA recorded more than double the SA incidents during the same period, in 2017 SA peaked higher than WA, after a dramatic dip in WA incidents of non-compliance since 2014. By 2019, incidents recorded by both jurisdictions were somewhat similar. In SA, the steady rise in noncompliance is consistent with the increased focus to target compliance in the fishery. SA instances again dropped in 2018 but rose slightly in 2019. Similarly, since 2017, WA recorded a steady increase of instances of noncompliance. In 2019, WA recorded only a slightly higher number of noncompliance.

**Fig 2:**
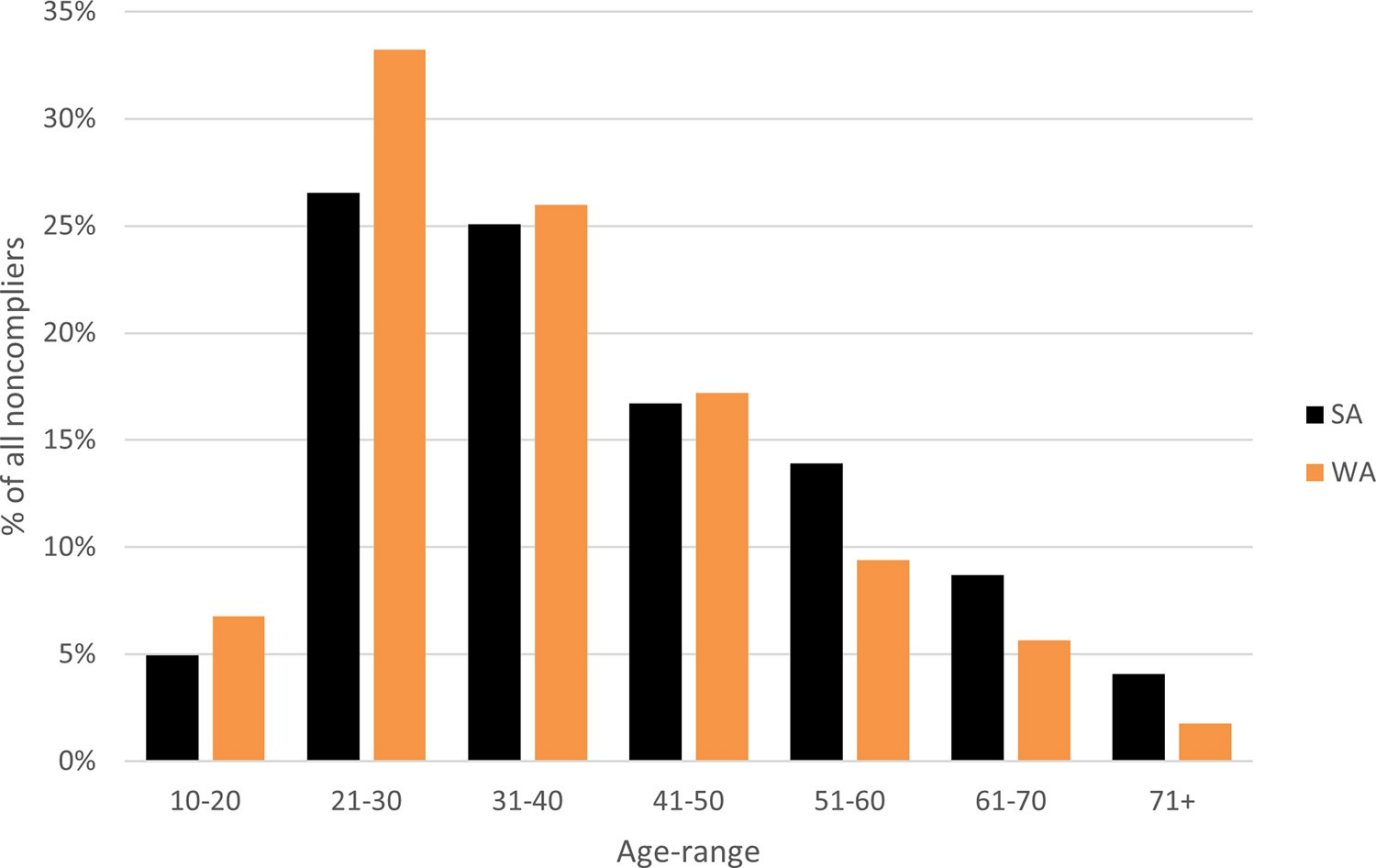
Percentage of instances of noncompliance (all) between 2009 and 2019 in Western Australia and South Australia by age-range of noncompliers.

Fig 2 shows the non-compliance age cohorts for WA and SA blue swimmer crab fishers. In instances where demographic data for fishers is not readily available, the age-range of noncompliers can usefully assist regulators to describe and target potential noncompliance among fishers. However, recreational fishers often fish in groups and this metric relates to only those identified as noncompliers within the group, rather than all participating fishers.

In WA and SA, noncompliers tend to be younger; in WA, two-thirds (n = 4,441, 66%) of noncompliers intercepted below 40 years of age and SA recorded similar results for the same age cohort (n = 1,780, 57%). WA recorded a marginally higher number of noncompliers in the 21–30 year age-range compared to SA (33% and 27%, respectively), while SA only exceeded WA noncompliers among the older cohorts (over 51 years) (combined totals 27% and 17%, respectively). Recreational participation in these fisheries requires little gear and experience, lending itself to wide participant engagement. Gender is another potential identifier to understand noncompliance. Overwhelmingly in both WA and SA, males were over-represented in the data.

In WA, males represented 77 percent (n = 5,208) of incidents of noncompliance between 2009 and 2019. Females in WA were attributed to 23 percent (n = 1,516) of incidents of noncompliance. Meanwhile in SA over the same time period, the breakdown of noncompliance incidents were even more likely to be attributed to males (n = 2,606, 83%) compared to females (n = 514, 16%) and 27 incidents (1%) failed to record the gender of the noncomplier.

Fig 4 breaks down the instances of noncompliance by month, aggregating across the 11 years between 2009 and 2019.

As the recreational catch method for these fisheries involves land-based wading and a need to see crabs on the bottom, it is understandable that greater numbers of fishers would engage in these fisheries over the spring and summertime months (September through February), whether legally or illegally. Overall, in SA the months of greatest noncompliance are September through January, with the data indicating that September had the highest number of offences (n = 729), whereas in WA, during November through March the amount of detected illegal activity is highest, peaking in January (n = 2,468). However, in WA, there are often undersize crabs early in the season (late spring) as they have yet to molt into their new, larger shell. There is therefore a high chance of catching undersize crabs during November and December.

Conversely, in both WA and SA, there is a dramatic decrease in the number of detected offences during May through August. This is due to several reasons: the number of patrols hours are lower (the 2018 and 2019 WA average May through August = 323 hours, compared to 671 hours September through April; the 2018 and 2019 SA average May through August = 28.5 hours, compared to 178 hours September through April); weather conditions are poorer, and the size and availability of crabs are lower, proving less desirable, and so fewer people travel to participate in the fishery. The WA fishery is generally closed from September through November to protect juvenile crabs and decrease the need for a large enforcement presence to check crab catches. In the southern hemisphere, in May through August, the daylight hours are shortest and are more likely to be cool, wet and windy and as such fisher effort (see catch method above) decreases. Crabs typically mate in autumn, and egg-carrying females are not allowed to be taken, further reducing the participation in the fishery over winter.

Fig 5 provides a day of the week snapshot of noncompliance.

This research considers only the recreational participants that were inspected in these blue swimmer crab fisheries. Greatest (noncompliance) activity occurs across the weekend days in both jurisdictions when working-age adults are less likely to be at work. In both WA and SA, detected offences are more than double on Saturday and Sunday (higher than 25% of all inspections) than weekdays (less than 15% of all inspections), typical of the workweek. Friday noncompliance increases, indicating that there is more than likely greater participation in the early evening (Fig 6). In WA, crab fishing is a popular family pastime, leading to greater participation on Friday through to Sunday. It is not possible to determine with our dataset whether we are seeing increased illegal activity, or simply higher fisher participation.

Fig 6 shows the breakdown of noncompliance instances across 2009 and 2019, by timeslot for both WA and SA.

The data show that in WA illegal activity is more commonly detected in early to later evening (16:00–22:00), whereas in SA, detected illegal activity corresponds to between midday and 14:00. This vast difference between the two jurisdictions can be explained by participation rates peaking over this period in each jurisdiction.

Few instances of noncompliance were identified overnight between midnight and 6:00, however this may align with decreased patrols during those times. Patrolling regulatory officers are shift-workers in WA and so can be scheduled to patrol across all times of day based on perceived risk and a need to promote *general* and *specific* deterrence.

Fig 7 shows noncompliance activity against patrol hours. These data show the general correlation between detected offences and patrol effort.

Across both WA and SA, as expected there is a correlation between increased patrolling hours and noncompliance intercepted. The WA patrolling strategy appears to have adjusted slightly between the three years. In 2018, the hours of patrol in WA totaled 5,788, increasing in 2019 to 7,543 hours spread across the year. Despite the change in hours of patrol, the rate of offences intercepted varied little. The patrol effort to noncompliance incidents intercepted averaged at six percent across both years.

This differs in SA. SA patrol data was not collected prior to July 2017. In 2018, 1,124 patrol hours were conducted increasing to 1,960 hours in 2019. While the hours of SA patrol were much lower than in WA, the patrol effort to noncompliance incidents intercepted averaged at 21 percent across the two years. This strategy of lower patrol coverage appears to lead to greater rates of noncompliance interception compared to hours of patrol.

Patrolling officers looking for various types of noncompliance, outlined in the overarching legislation for WA and for SA outlined in Table 1. Figs 8 and 9 disaggregate the total offences between 2009 and 2019 by type of noncompliance for WA and SA, respectively.

Overwhelmingly, the majority of noncompliance in WA and SA relates to possessing undersize crabs, as shown in Figs 8 and 9 (WA: n = 4,884, 76%; SA: n = 2,325, 81%). Fishers must gauge crabs to determine whether they can legally keep them. Suitable gauges are readily available, although many fishers risk using everyday objects as gauges. The next most frequently recorded type of noncompliance in both WA and SA was exceeding the allowable daily catch (or bag) limit (WA: n = 872, 13%; SA: n = 201, 7%). In WA, 177 (3%) instances of noncompliance involved a failure to have a license, because a license is required if the catching of crabs made use of a boat.

While the excess take of crabs was not the highest category of noncompliance type overall, excess take can have great ecological impact on stock estimates and the management of these fisheries (including imposing longer recreational closed seasons and lower daily limits). Both WA and SA experienced excess crab take, see Fig 10.

For both WA and SA instances of noncompliance relating to excess crab take, the majority of noncompliers took between one and 10 crabs over the daily limit (Fig 10). Specifically in WA, of the 872 instances of noncompliance, 345 (40%) involved 10 or fewer crabs over the daily limit. However, the next highest group took more than 30 crabs (n = 222, 25%). This group represents a quarter of all noncompliers who took any number over the daily allowable catch limit.

In SA, of the 201 instances of exceeding the daily limit, more than half (n = 108, 54%) took 10 or fewer additional crabs.

Responding to noncompliers in WA and SA takes similar forms, as instructed by the legislation. From least to most harsh, noncompliance is dealt with through a series of sanctions including warnings/cautions, infringements/expiations or through prosecution (brief). Figs 11 and 12 show the proportion of outcomes for WA and SA, respectively, by year between 2009 and 2019.

In WA in 2009, infringements (n = 109, 50%) were issued more frequently than warnings (n = 75, 34%) and sharply dropped below the frequency of warnings issued, before rising again in 2017 (n = 189, 56%) before warnings and infringements leveled out in 2018 and 2019. Overall in WA, a greater proportion of warnings were issued compared to infringements between 2009 and 2019 (n = 3,388, 55%; n = 2,407, 39%, respectively). Over the 11-year period, prosecutions experienced a slight downward trend.

SA recorded a very similar number of cautions and expiations between 2009 and 2019 (n = 1,437, 50%; n = 1,438, 50%, respectively). Similar to WA, SA recorded a higher number of expiations in 2009 compared to cautions (n = 120, 83%; n = 24, 17%, respectively). Over the course of the following decade, the recorded incidents of cautions increased while expiations decreased, despite minor fluctuations in 2014 and 2017. In 2019, the trajectory shows a decrease in expiations (n = 145, 34%) and increase in cautions (n = 281, 66%). Across the time period, Fig 12 shows that there is a strong downward trend in expiations while simultaneously, there is a strong upward trend in cautions, despite minor fluctuations. The decrease in expiations in South Australia appears to be driven by the decrease in possession of more than 10 undersize crabs’ offences, while the increase in cautions appears to be driven by the increase in possession of 0–10 undersize crabs’ offences. Through the same period of 2009 to 2019, the rate of prosecutions remained static.

In WA and SA, formal warning notices and infringement notices / expiations (involving a monetary penalty) and prosecutions seeking monetary penalties, property forfeitures and sometime license suspensions are applied for noncompliance in these fisheries, rather than, for example, periods of incarceration. Fig 13 provides an overview of the monetary penalties applied in WA and SA between 2009 and 2019.

Fig 13 highlights a differing strategy of involving monetary penalties between the two jurisdictions. WA favors monetary penalties over A$1,000 (n = 244, 67%) while SA favors penalties up to A$500 (n = 1,270, 88%). Overwhelmingly, SA handed down more monetary penalties compared to WA (n = 1,438; n = 364, respectively). Relevantly, the proportion of penalty amounts in each jurisdiction stayed relatively consistent over the time period of analysis.

### Discussion

The results from the anonymized regulator data analyzed, provide useful trends over the 11-year period. In addition to insights from each fishery, comparisons are drawn between the WA and SA fisheries that may be useful in managing the fisheries. Interpretations from these results against deterrence theory also provide an evidence-base to inform regulatory strategies within and beyond these fisheries and jurisdictions.

For licensed fisheries, participant demographic data is collected, including gender, age, and address, among others, however as these data relates to a mainly license-free fishery, information is not collected about recreational participants, unless an incident of noncompliance is recorded. As such, these data in aggregate provide the most reliable overview of participants and their engagement in these fisheries. It is important to note, these data do not necessarily provide a representative sample of the participants, whether compliant or noncompliant. Regardless, in the absence of any other participant information from recreational surveys, it provides useful insight into those participating in these fisheries.

Through the data analyzed, interpretations can maximize the opportunity for regulators to enhance compliance through community education and targeted enforcement activities. Optimizing compliance facilitates a balance between regulator cost and effort, and community or in this case, fisher, engagement. Understanding the fishery through data analysis can assist in determining how best to allocate limited regulator resources. While operational regulators may already have a strong sense of how best to appropriate resources, providing an evidence-base satisfies policy- and other decision-makers, as well as taxpayers. The regulatory philosophy of the fishery involves increasing participant compliance through greater lower-cost education rather than increasing costly enforcement via patrols. This philosophy aligns with *deterrence theory*. Following a *general deterrence* strategy, trends on noncompliance activity recorded (rather than focused on the specific noncompliers) yields informative lessons that can be applied to enhance the effectiveness of education and enforcement strategies applied to encourage compliance within these fisheries. Against deterrence theory, several lessons can be learned to support regulators making evidence-based decisions about their education and enforcement strategies. The three elements of deterrence theory, *swift*, *certain* and appropriately *severe* can each be considered against the data presented in the previous section. Warning and infringement notices are swiftly issued and serve to support deterrence theory. Based on the available data, the *swiftness* of responses cannot be analyzed, and no further interpretations can be drawn; *certainty* and *severity* can.

It should be noted that both regulators are resource limited and so all observed offence data is a subset of actual non-compliance that occurs when enforcement staff are not present (although public reporting of illegal activity can mitigate this gap). When dealing with enforcement data, without independent data relating to participation in fishing activities, it is always possible that the enforcement data could be biased towards times of peak illegal activity. Or it could be possible that enforcement patterns are widely known, resulting in under-representation of non-compliant activity in the enforcement activity. Total illegal catch, and total amount of illegal activity are unknown and cannot be estimated without some idea of fisher participation or catch. We have taken the simple assumption that the data are representative of fishing activity and non-compliant activity in both fisheries. This assumption highlights the importance of complementary independent data from fisher or creel surveys. Although such data are available for both fisheries, it has not been within scope of this study.

**Fig 3:**
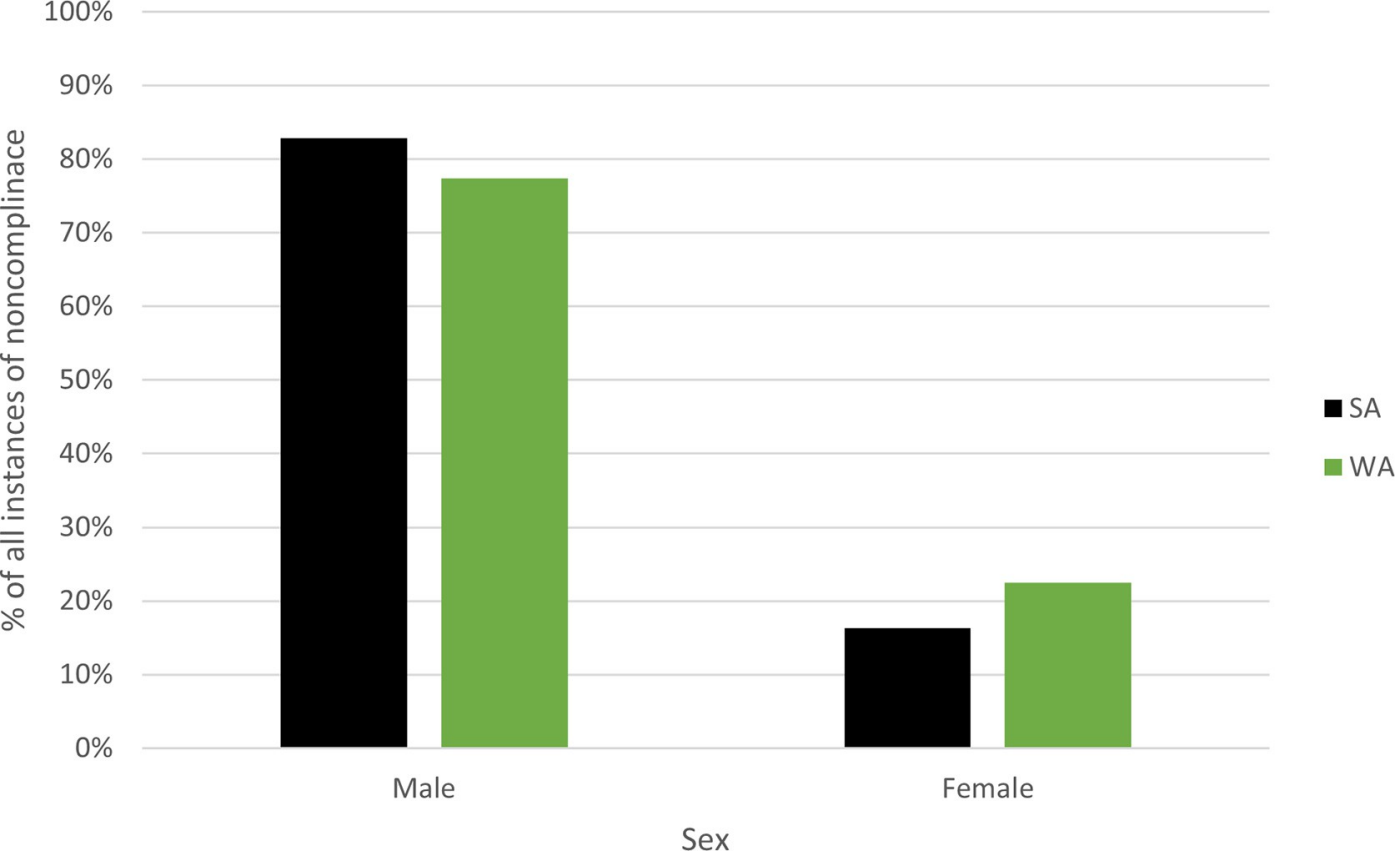
Percentage of instances of noncompliance (all) between 2009 and 2020 in Western Australia and South Australia by males and females.

Given that we do not know participation rates within these fisheries, it is difficult to assess the harm resulting from illegal activity. Understanding the full nature and extent of the fishing population would allow for more accurate profiling to assist regulators target potential noncompliers. Reflecting on the Results section, for example, males failed to comply far more frequently than females and fishers below 40 were among those least likely to comply (Figs 2 and 3). It is probable that the main cohort of fishers participating in these fisheries are males aged below 40 years; nonetheless, useful learnings from aggregate noncompliance data can support regulatory responses. Indeed, strategies of disseminating educational information, such as via various communication channels including social media, acknowledging that preferred social media platforms differ by age group, and installation of Quick Response (QR) codes on signage in common fishing areas may reach a broader audience by allowing fishers to follow a link that leads them to fishing rules in their language for instance. Whereas, given that a higher number of older, noncompliant participants exist in SA, in addition to educational communication via social media, forms of communication more familiar to older populations such as mail out and/or email may be more effective.

Additionally, as WA and SA recorded noncompliers below 20 years of age, disseminating fishery information as part of school education packages on environmental sustainability may enable multigenerational education when families subsequently engage in this recreational fishery. This educational strategy may be effective in achieving *general* deterrence.

**Fig 4:**
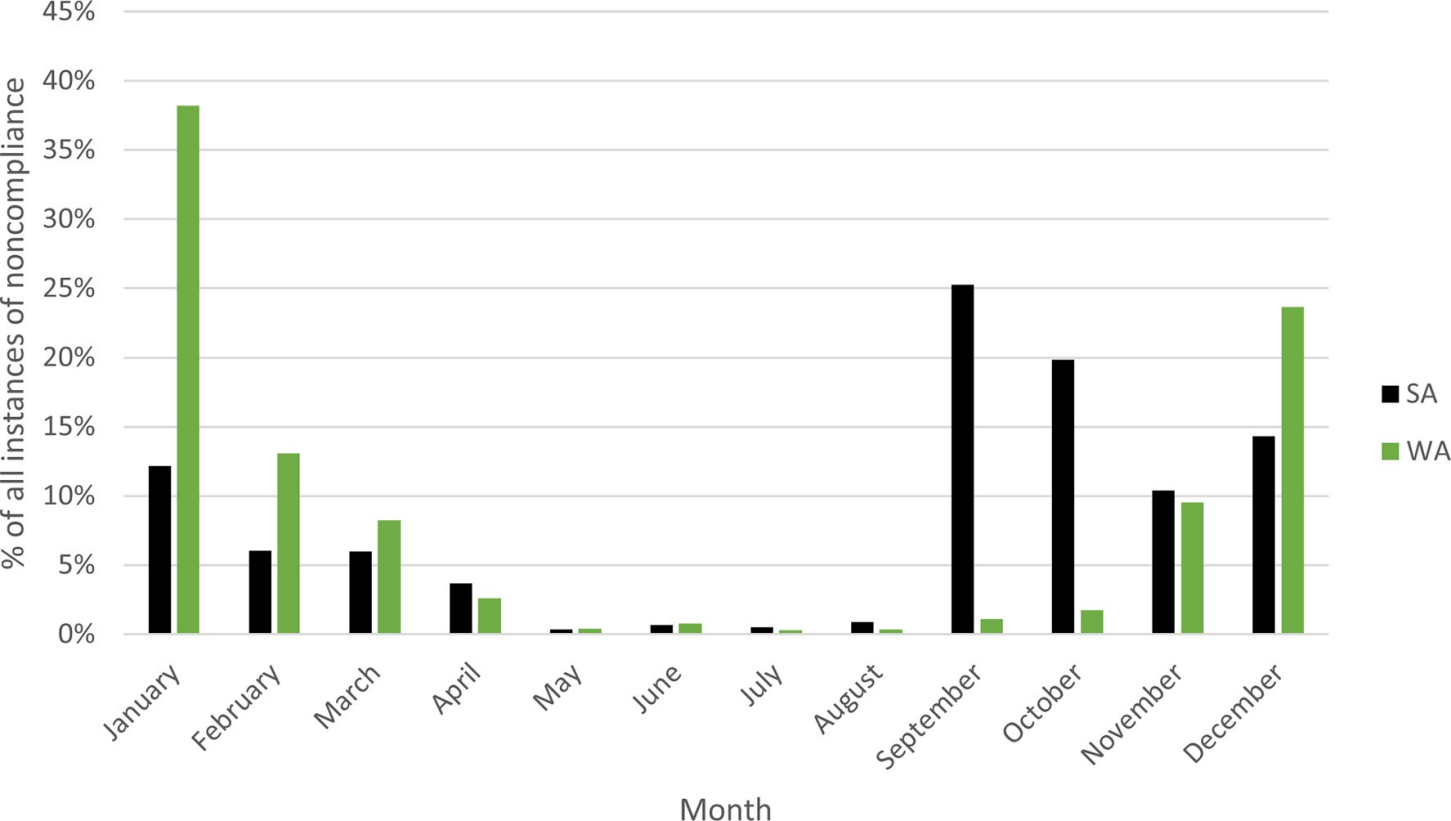
Percentage of instances of noncompliance (all) between 2009 and 2019 in Western Australia and South Australia by month.

**Fig 5:**
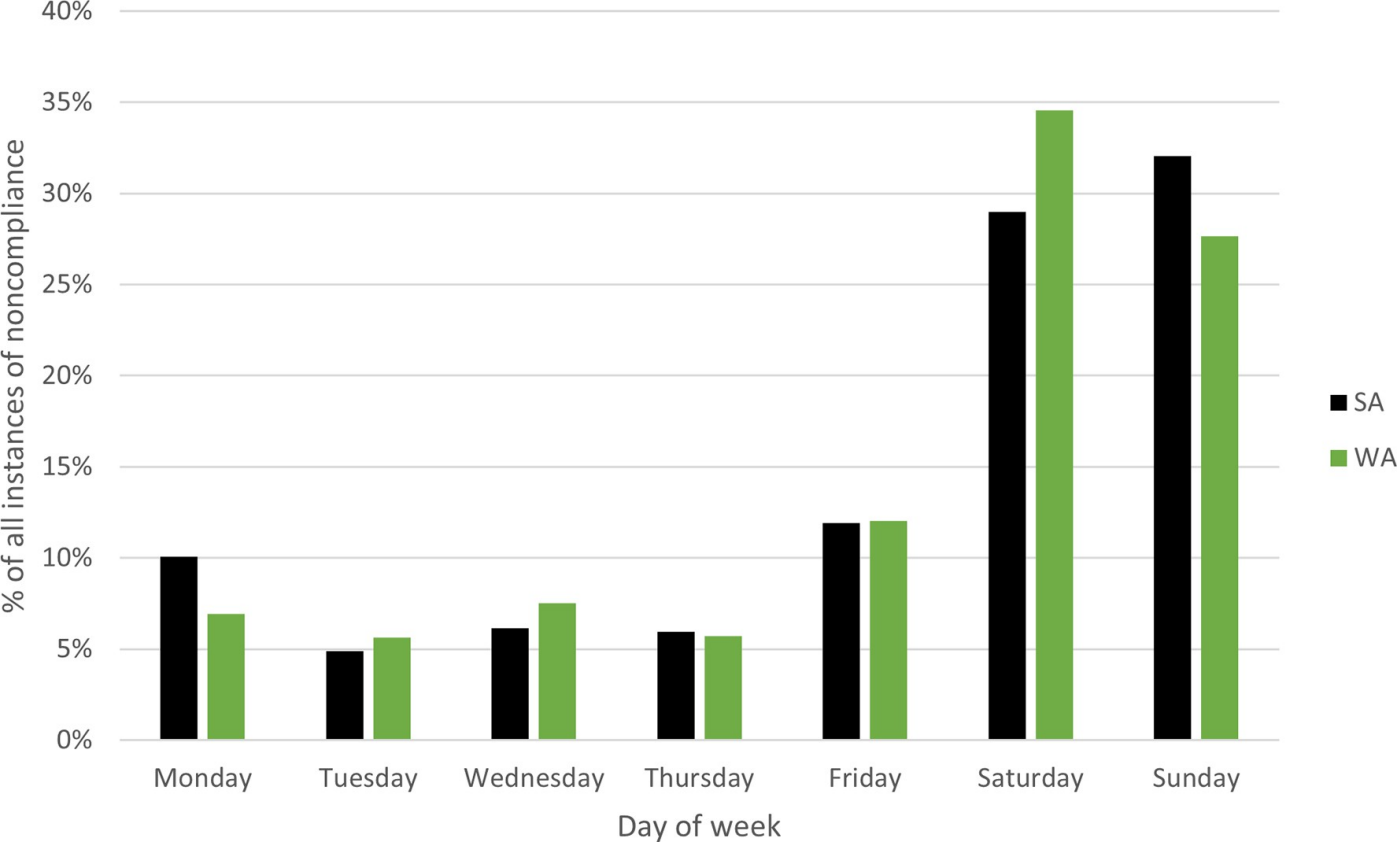
Percentage of instances of noncompliance (all) between 2009 and 2019 in Western Australia and South Australia by day of the week.

**Fig 6:**
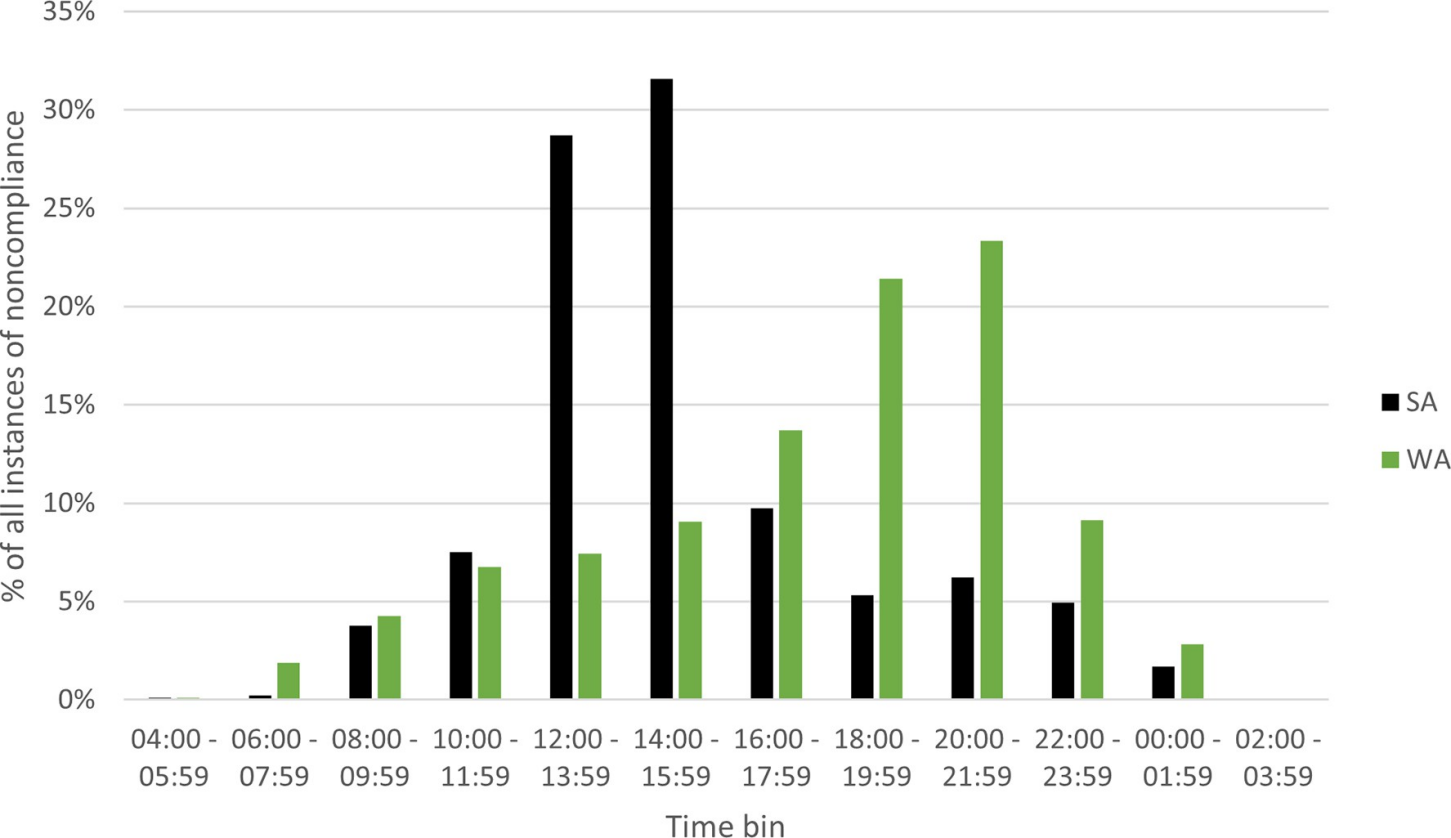
Percentage of instances of noncompliance (all) between 2009 and 2019 in Western Australia and South Australia by time of offence.

Having an understanding of the general profile of the offenders and when noncompliance is more likely to occur is necessary to ensure patrol efforts and noncompliance activity are aligned. During the southern hemisphere warmer months (between October and April) noncompliance incidents were more likely, with slight variations between the two jurisdictions (Figs 4 to 6). Noncompliance at the weekends was also more common, though the times of day differed between the two jurisdictions. As this dataset relates to a recreational fishery, greater participation at the weekend is expected, though without overall participation rates it is impossible to determine whether the rate of noncompliance per participant is higher at the weekend compared to any other time during the week.

**Fig 7:**
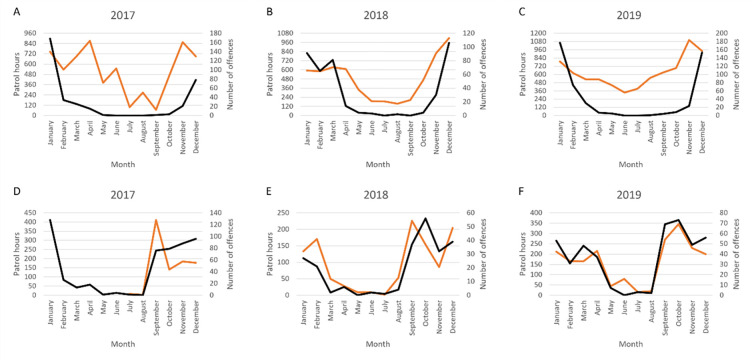
Number of all instances of noncompliance (black) and patrol hours (orange) per month in Western Australia for the years 2017 (A), 2018 (B), and 2019 (C), and South Australia for the years 2017 (D), 2018 (E), and 2019 (F).

Notwithstanding, regulatory efforts to intercept noncompliance can strategize targeted patrols based on this evidence of noncompliance, to ensure regulatory effort can be optimized (Fig 7). In addition to patrolling officers, other forms of notification may result in instances of recorded noncompliance, such as public reporting via *FishWatch* or as detected and reported by other government officials operating in the area. Common among prevention of other crime types, along with physical patrols in hotspot areas, other surveillance strategies may be utilized to limit the likelihood of noncompliance, for example increase signage of bag limits and open/closed seasons; increase bright street/area lighting; use of CCTV; increase passerby visibility from major access points, such as roads; encourage neighboring communities to report suspicious incidents.

Increased visibility of patrolling regulators and other forms of surveillance will deter would-be noncompliers, contributing to *general* deterrence, however motivated noncompliers are less likely to conduct their noncompliant activities during peak fishing periods when visibility is high, and instead opt for outlier times, such as between midnight and 6:00 (Fig 6). Harsher penalties may be considered as a method of *specific* and *severe* deterrence for noncompliers intercepted during these times as it implies clear motivation and if successful in evading interception, possible likelihood of recidivism. *General* compliance can be achieved not only through formal regulation. Rather, compliance through peer oversight, if *en masse*, participating fishers are educated of the overarching regulations, there is greater opportunity for compliance.

**Fig 8:**
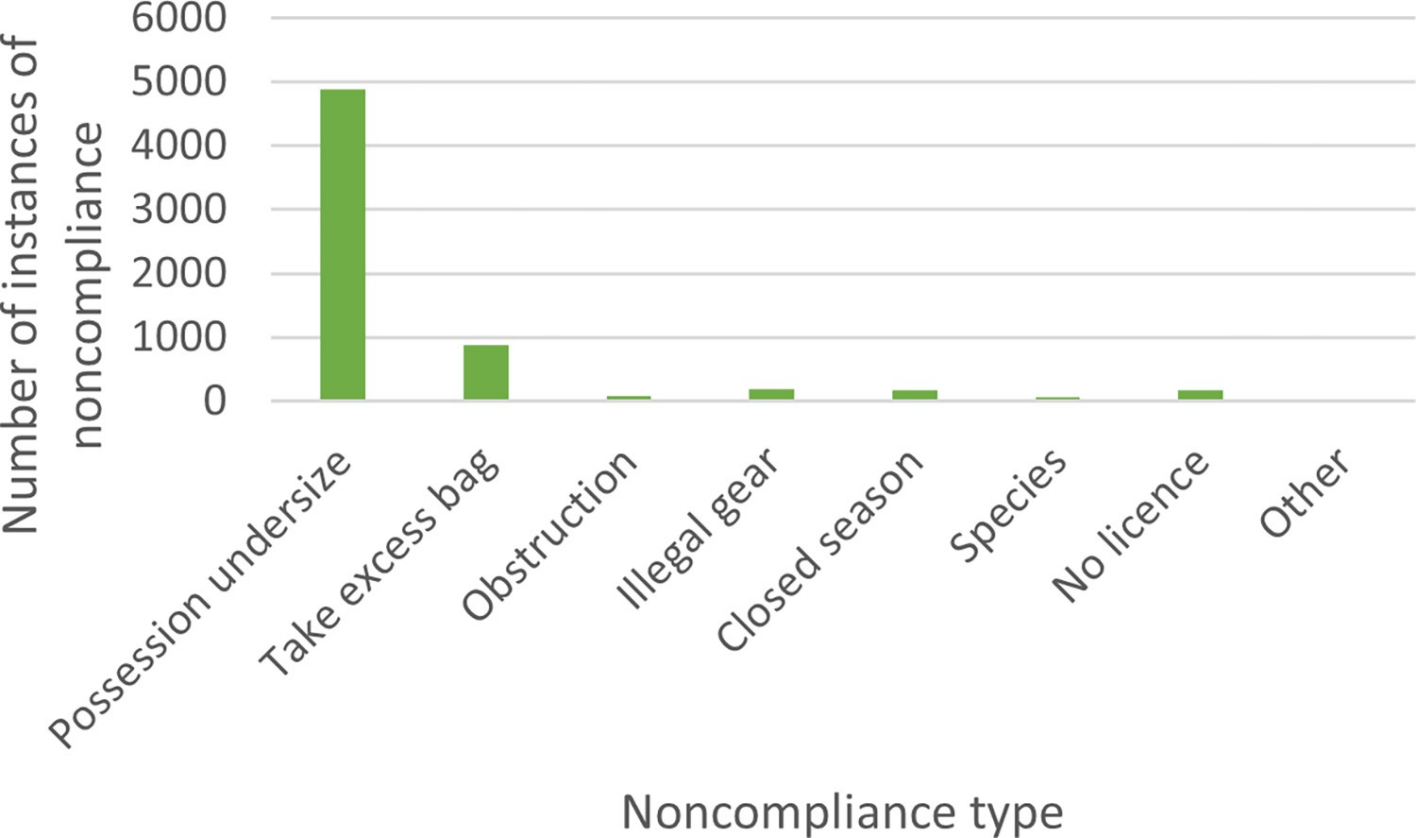
Total number of instances of noncompliance by type in Western Australia between 2009 and 2019 *(left)*.

**Fig 9:**
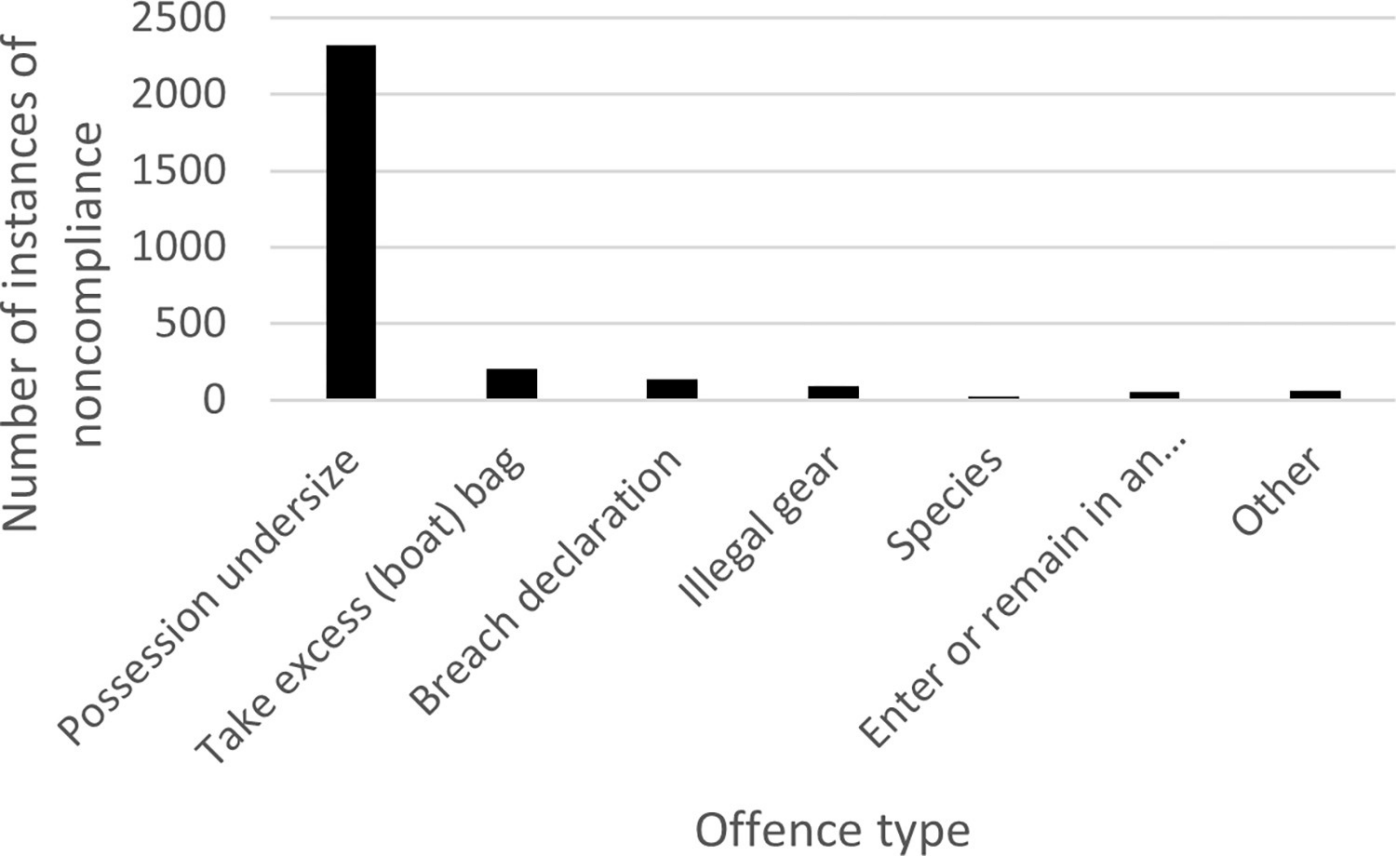
Total number of instances of noncompliance by type in South Australia between 2009 and 2019 *(right)*.

**Fig 10:**
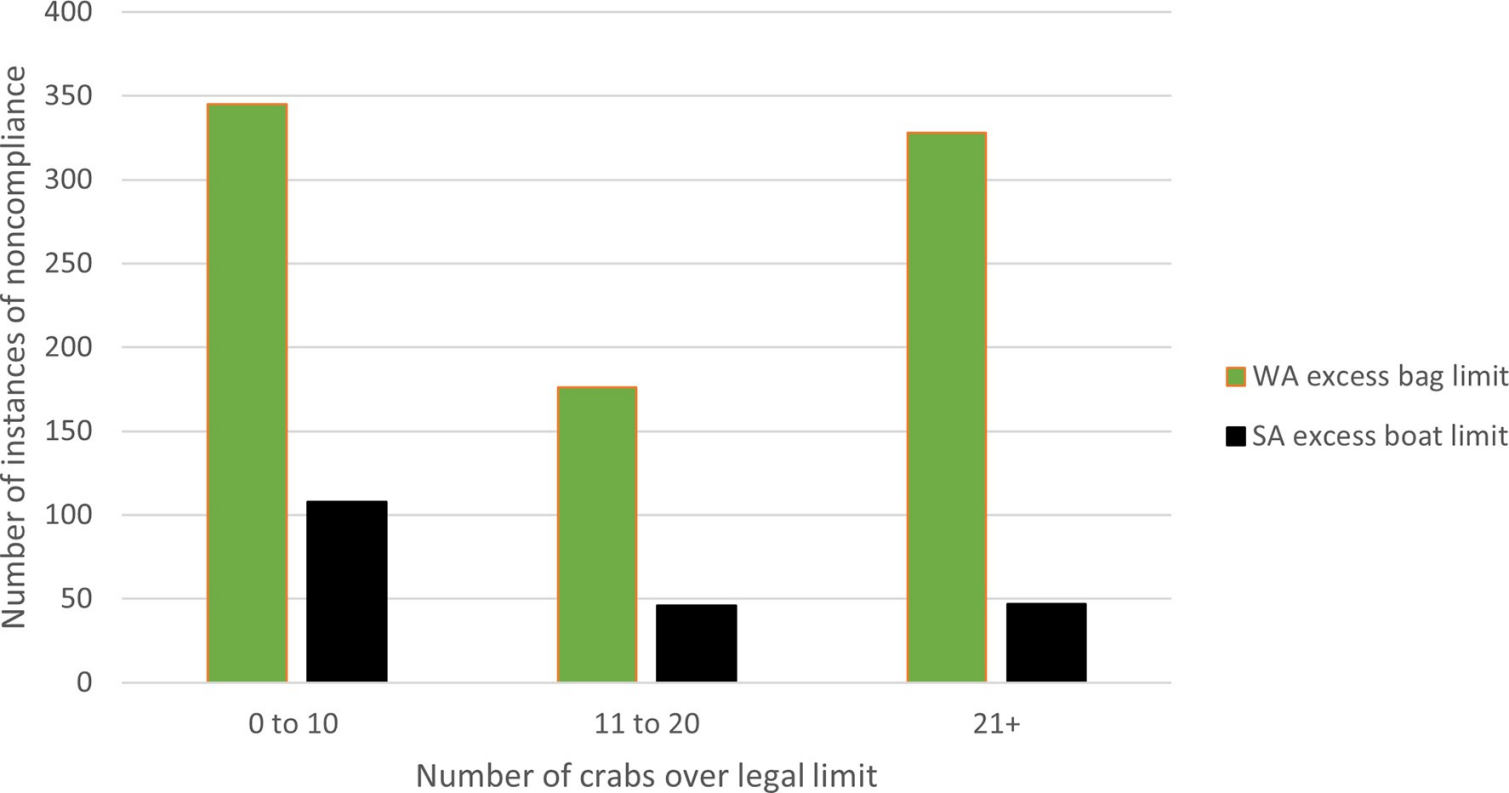
Total number of excess land bag (WA) and excess boat bag (SA) offences by number of crabs above legal bag limit between 2009 and 2019.

Figs 8 to 10 show the types of noncompliance occurring. For regulators and policymakers, this can assist to determine the effectiveness of legislation and any relevant subsidiary laws and policies. It can also assist fishery managers in decision-making to sustain stock numbers. For example, WA closes the fishery to protect vulnerable crabs and minimize the requirement for large numbers of inspections over winter, whereas the SA fishery operates year-round. During the period of closure, patrols continue (Fig 7). While there may be ecological differences in the fishery, the management strategy to close the fishery *and* continue enforcement may be as effectively achieved through enforcement alone, as evidenced in SA. Conversely, the financial cost to the regulator to close the fishery may be minimal and incidents of noncompliance also low (May to October total incidents of noncompliance: n = 356, 5%), therefore extending the closure may reduce undersize take during the open season. Closed seasons are effective strategies given they are low cost and can utilize patrols operating for other fisheries.

Drawing on deterrence theory, the *certainty* of interception can be determined from factors such as patrolling hours; and zero tolerance for undersize catches. Compliance by fishery participants depends on unambiguous messaging from the regulator. Public conveyance of rules and penalties must be part of the education communication strategy.

Preventing future noncompliance can be best achieved by imposing suitable deterrents aimed to dissuade the general community at large, and *specific* deterrence to dissuade the individual [40]. Commonly, new and/or harsher laws and penalties are imposed to achieve *general* deterrence [42]. Arguably, while this may be effective for some crimes, it also requires an increase in law enforcement resources to effectively police. This regulatory over-presence may reduce the enjoyment of the fishing experience, which may be unfeasible. For these fisheries, regulatory measures must instead be weighted to cost criminals more than they seek to benefit, while still considering the public interest in imposing harsh penalties.

**Fig 11:**
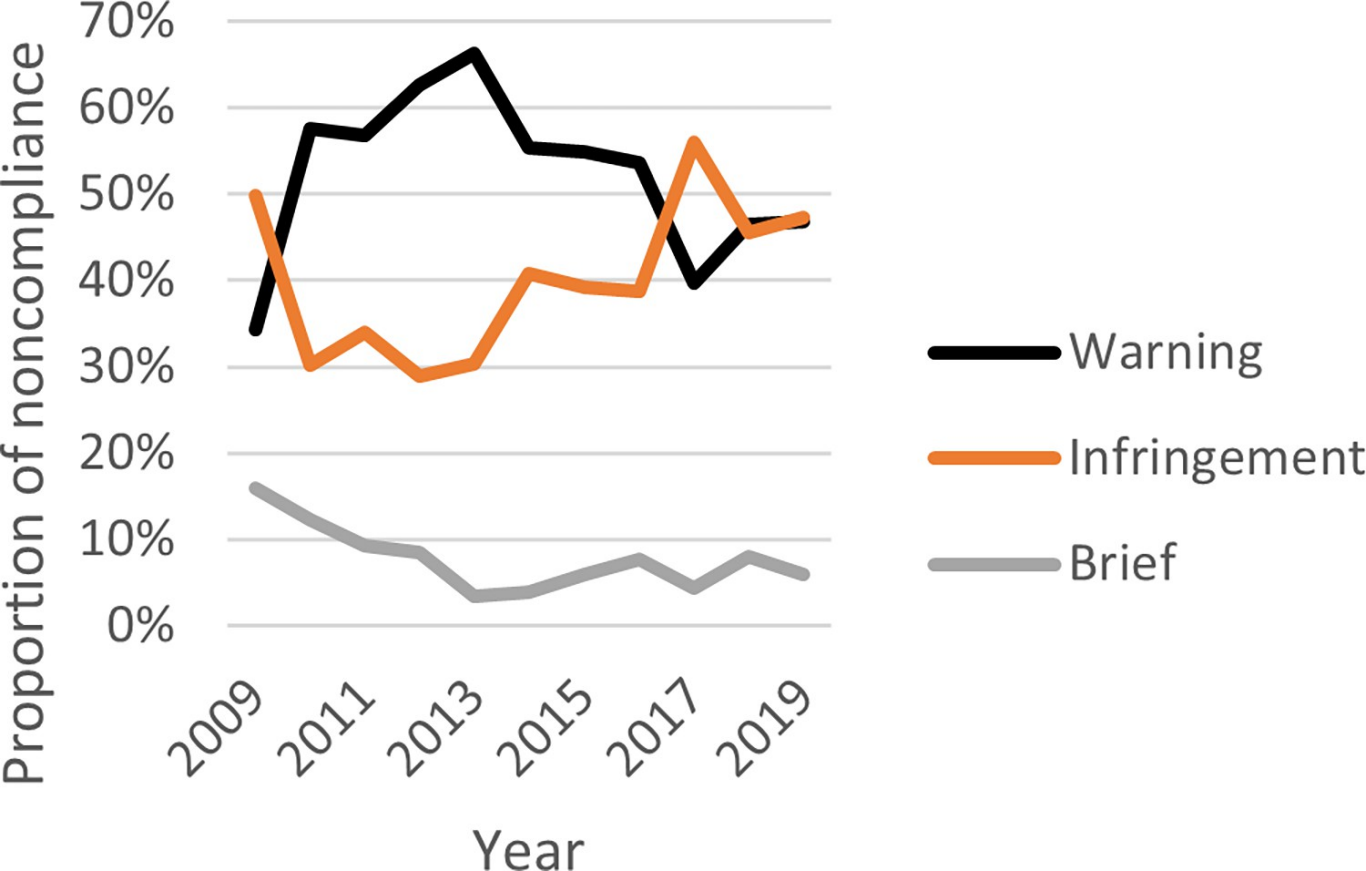
Proportion of different offence outcomes in Western Australia over time (2009–2019) *(left)*.

**Fig 12:**
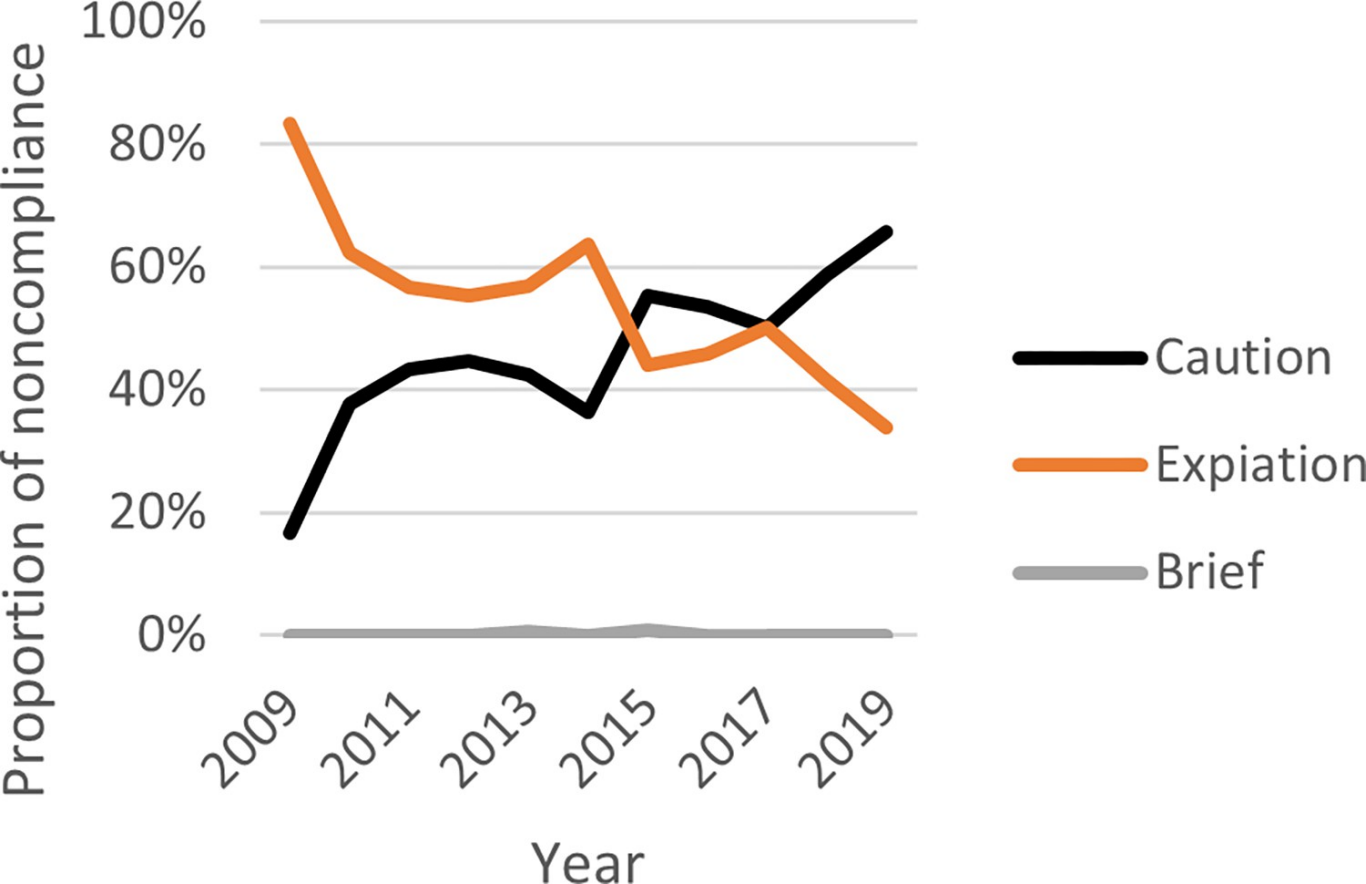
Proportion of different offence outcomes in South Australia over time (2009–2019) *(right)*.

**Fig 13:**
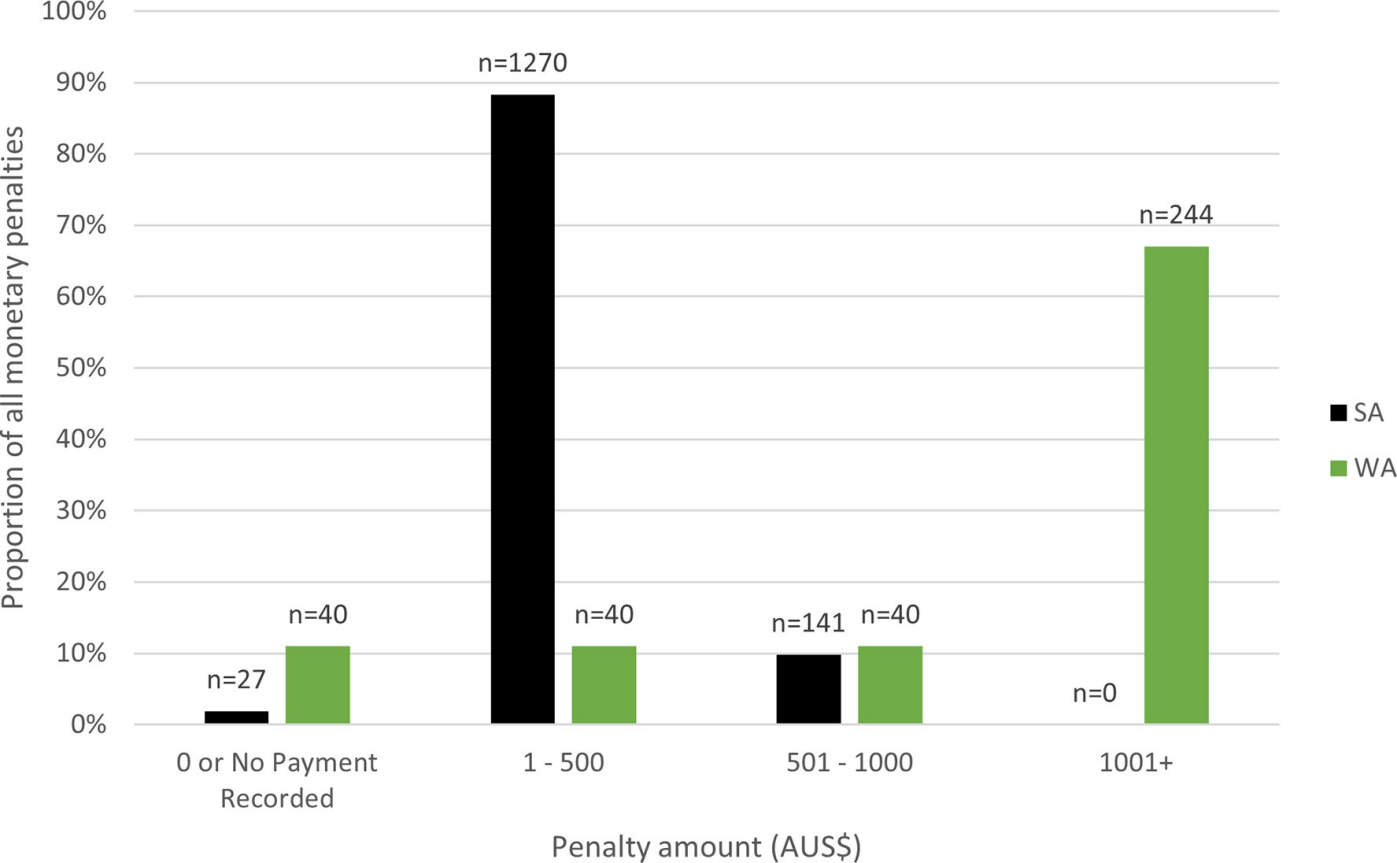
Proportion of different penalty amounts for all monetary penalties in Western Australia and South Australia between 2009 and 2019.

How *severe* penalties are relates to the likelihood of warning/caution; infringement/expiation; or prosecution (brief). Both WA and SA regulators show greater use warning/caution over any other form of penalty [see Figs 11 and 12]. If an infringement/expiation is applied, how high the monetary penalty applied might be a suitable deterrent. Strategies to apply penalties to noncompliance differs between the two jurisdictions, in that WA applies harsher monetary penalties per noncomplier than SA, however SA hands down a greater overall number of monetary penalties [see Fig 13]. The WA strategy aligns well with the *specific* deterrence approach and is suitably *severe* (an element of deterrence theory) whereby the cost for an individual noncompliers in receipt of a high monetary penalty would outweigh the benefit. In contrast, the SA strategy aligns with the *general* deterrence approach, and is suitably *certain* (an element of deterrence theory), whereby notifying would-be noncompliers their wrongdoing would be highly likely to attract a monetary penalty. Relevantly, despite SA handing down more monetary penalties than WA, in aggregate, SA collected a total of $305,688 in penalties, while WA collected $702,386 in penalties. Both of these strategies have merit. Being aggregate, anonymized noncompliance datasets, it is not possible to track recidivists in these fisheries to truly understand the deterrent impact.

Despite the results being unable to show recidivism, regulators would nonetheless be prudent to strategize the overall impact of a high number of minor noncompliers compared to a low number a serious noncompliers, as the aggregate result of a low number of serious noncompliers could be as harmful, or even more harmful. This may assist in determining the appropriate use of sanctions, such as monetary penalties to achieve *specific* deterrence, or management strategies such as seasonal closures to achieve *general* deterrence.

Through the analysis of these data, it is clear that the regulatory responses adopted by each jurisdiction yield differing outcomes. Given that the blue swimmer crab fisheries in WA and SA are license-free fisheries, regulators only interact with, and record activity of, recreational participants in these fisheries relating to noncompliance. As is the case with other fisheries, fishers exceed patrolling officers, not all noncompliance instances are intercepted, meaning it is challenging to have a clear understanding of catch, both legal and illegal. Further data to estimate catch, and establish recreational (and commercial) catch limits aligned with sustainability is needed. Responses to encourage compliance is sensible and research to collect recreational fisher perceptions continues to contribute to this gap in knowledge.

## Conclusions

Effective fisheries noncompliance management can be expensive and may reduce fisher enjoyment, therefore finding a balance between education and enforcement while sustaining the fishery is important. This research analyzed data held by Australian fisheries regulators DPIRD (WA) and PIRSA (SA) revealing informative trends in each blue swimmer crab fishery that seek to shape educational and enforcement approaches to these fisheries.

The results of the datasets analyzed provide two important outcomes: first, through the analysis of these data, emergent trends provide an evidence-base otherwise absent from the existing regulatory frameworks applied within these jurisdictions. Comparisons between the two jurisdictions can also assist where there are stark differences in approaches to noncompliance management strategies (e.g. application of seasonal closures, patrolling hours, financial penalties). These results have potential application within and beyond these fisheries and jurisdictions.

Second, the application of deterrence theory confirms the usefulness of a multi-disciplinary approach to analyze and interpret noncompliance fisheries data. This can inform social-scientific approaches to noncompliance educational and enforcement strategies can be reliably based to ensure relevant populations and activities can be appropriately targeted.
